# Staphylococcal Scalded Skin Syndrome in a Newborn

**DOI:** 10.4103/0974-777X.52981

**Published:** 2009

**Authors:** D Jeyakumari, R Gopal, M Eswaran, C MaheshKumar

**Affiliations:** *Department of Microbiology and Paediatrics, Madagadipet, Pondicherry, India, Affiliated to Pondicherry University*; 1 *Department of Sri Manakula Vinayagar Medical College and Hospital, Madagadipet, Pondicherry, India, Affiliated to Pondicherry University*

**Keywords:** *Staphylococcus aureus*, Scalded skin syndrome, Exfoliative toxins

## Abstract

A six-day-old newborn was admitted with exfoliating erythematous lesions over the face, of two days duration. The lesions spread to the rest of the body during the next two days. A diagnosis of Staphylococcal Scalded Skin Syndrome (SSSS) was made clinically and confirmed by isolation of *Staphylococcus aureus* from a blood sample. The child responded to Injection vancomycin and no fresh lesions were seen after the next 48 hours. However the child developed severe pneumonia and left against medical advice.

## INTRODUCTION

Staphylococcal Scalded Skin Syndrome (SSSS) is an extensive exfoliative dermatitis that occurs primarily in newborns and previously healthy children. This syndrome is caused by Staphylococcal exfoliative or epidermolytic toxin produced by *Staphylococcus aureus*. The severity of the disease varies from being a localized skin lesion to a more extensive generalized condition, characterized by cutaneous erythema followed by profuse peeling of the epidermal layer of the skin.[1] The duration of the disease is brief with a low mortality rate among children with prompt treatment. A case of SSSS in a newborn is reported because of its rare occurrence.

## CASE REPORT

**Figure 1:**
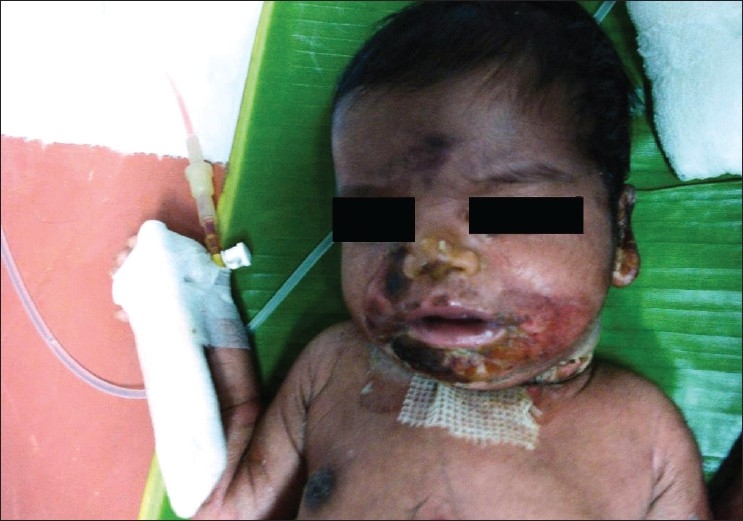
Lesions over the face and neck

**Figure 2:**
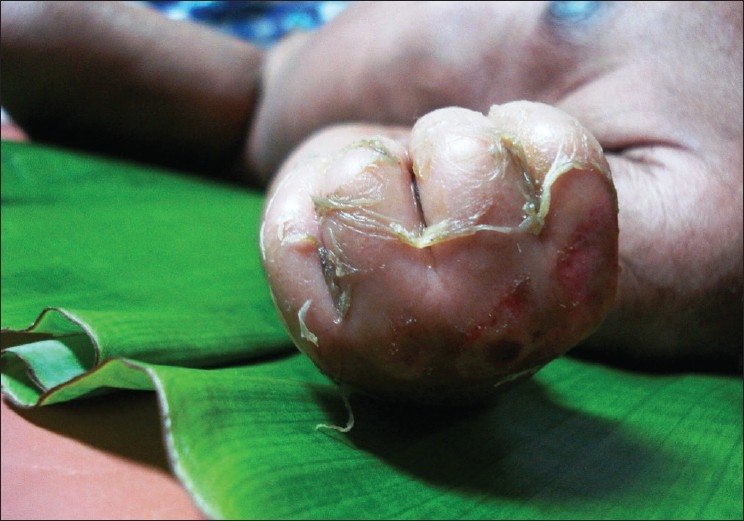
Lesions over the back of the hand

**Figure 3:**
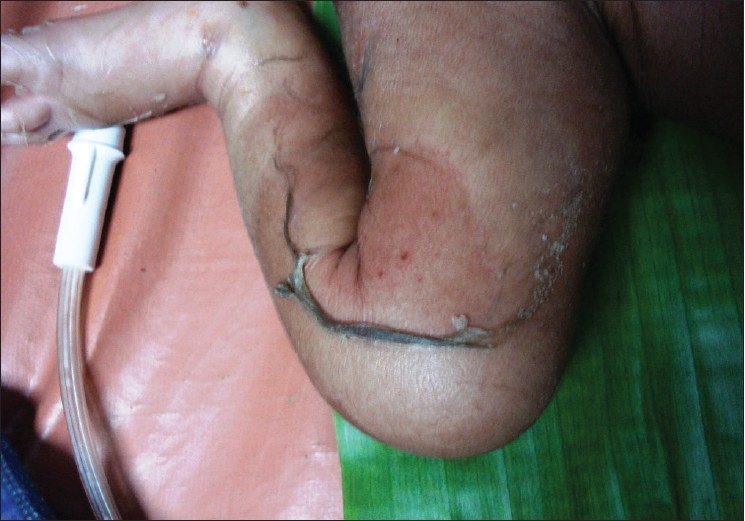
Skin peeling over the thigh

**Figure 4:**
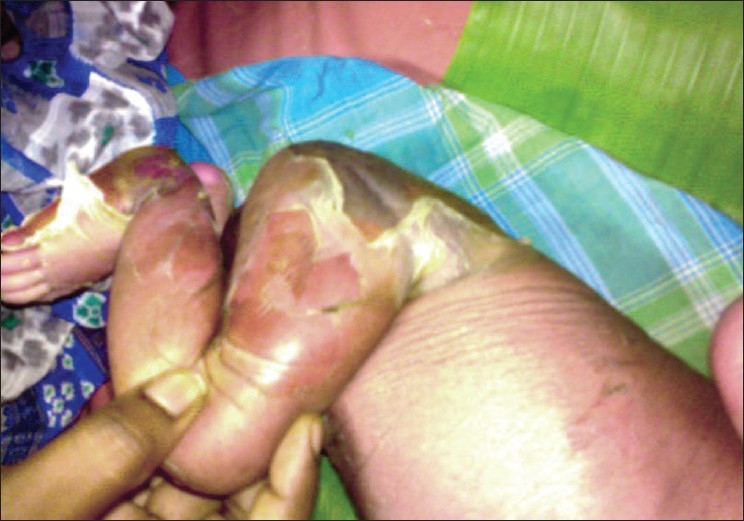
Extensive exfoliations over the gluteal region

**Figure 5:**
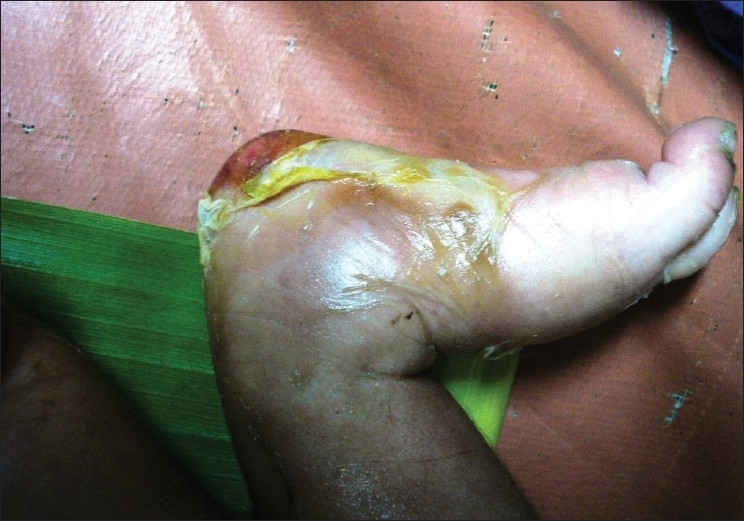
Secondary infections over the right foot

A six-day-old newborn was admitted with erythematous lesions over the face, of two days duration. The child was a full-term, normal baby. The child developed an erythematous rash on the fourth day of birth. The lesion started behind the ear and spread to other parts of the face and neck within the next two days. There was no history of administration of any drugs after birth. On examination at admission the child was toxic, febrile with erythematous lesions around the mouth, nose, and over the neck [Figure 1]. The oral mucosa was normal. The lesions spread to the hands, gluteal region, thighs, and legs over the next two days [Figures 2–4]. A culture of the blood sample collected on the day of admission yielded a pure growth of *Staphylococcus aureus*, which was sensitive to oxacillin, cefazolin, cefatoxime, vancomycin, netilmycin, ticarcillin, and ciprofloxacin. Swabs collected from the cutaneous lesions on the day of admission did not yield any growth. In view of the extension of the lesions to other areas of the body the child was put on injection vancomycin and topical soframycin in addition to cefatoxime started earlier. A clinical diagnosis of Staphlococcal Scalded Skin Syndrome (SSSS) was made. The lesions healed over the next two days and no new lesions were observed. The child became afebrile and was feeding well. However the child developed tachypnea with a respiratory rate of 76/minute on the sixth day of admission and had an episode of generalized seizures, which responded to intravenous calcium gluconate. An X-ray of the chest was suggestive of right upper lobe pneumonitis. An ECG showed sinus tachycardia with right ventricular hypertrophy. Repeat blood culture on day six of admission was sterile. The patient developed a swelling over the right ankle on day seven [Figure 5]. Aspirated pus yielded coagulase-negative Staphylococcus. A nasal swab of the mother yielded a pure growth of *Staphylococcus aureus*.

## DISCUSSION

Staphylococcal Scalded Skin Syndrome describes a spectrum of superficial blistering skin disorder caused by the exfoliative toxins of *Staphylococcus aureus*. In a severe form, the exfoliation can spread to cover the entire body surface. [2] SSSS particularly affects infants and young children. Lack of protective antibodies to exfoliative toxins A and B (ETA and ETB) and immature renal function, which impairs the ability to excrete the toxin have been suggested as reasons.[3] In infants and young children, potentially fatal complications include hypothermia, dehydration, and secondary infections.[4] Three forms of the Staphylococcal skin disease have been described in neonates, namely, SSSS, Bullous Impetigo (BI), and Staphylococcal Scarlet fever. SSSS and BI have many features in common. However, compared with BI the skin lesions of SSSS are larger, *Staphylococcus aureus* is less frequently isolated, and less inflammatory infiltrate in the skin lesions is noticed. Characteristically SSSS consists of diffuse erosions with epidermal separation in the submucosal layer through the granular layer. The differential diagnosis of SSSS includes drug-induced toxic epidermal necrolysis, epidermolysis bullosa, bullous mastocytosis, herpetic lesions, and neonatal pemphigus.[5] Clinically the SSSS mimics toxic epidermal necrolysis (Lyell's syndrome). The typical features of SSSS are involvement of periorificial face, de-epithelialization of friction zones, and absence of mucosal involvement. However, in toxic epidermal necrolysis (Lyell's syndrome) there is a severe involvement of visible mucosa and also the respiratory, gastrointestinal, and urinary tract mucosae.[5]

In the case reported, the characteristic skin lesions and failure to isolate *Staphylococcus aureus* from the lesions, but from blood culture, absence of any mucosal lesions, and no history of administration of drugs was suggestive of SSSS. With prompt treatment the progression of the lesions was arrested and no new lesions occurred. In the case described the child responded to injection vancomycin with no new lesions and became apyrexic within 48 hours. However, the child subsequently developed arthritis and pneumonia probably due to secondary infection. The child was discharged on request by the parents and was lost to follow up.

## CONCLUSIONS

Staphylococcal Scalded Skin Syndrome can occasionally lead to serious complications like pneumonia, septic arthritis, hypothermia, dehydration, and secondary infections. With appropriate management, however, mortality due to SSSS in children remains below 5% in comparison to about 60% in adults. Therefore, early diagnosis and appropriate treatment can prevent the mortality associated with these complications.
